# Oral Contraceptives Impair Complex Emotion Recognition in Healthy Women

**DOI:** 10.3389/fnins.2018.01041

**Published:** 2019-02-11

**Authors:** Rike Pahnke, Anett Mau-Moeller, Martin Junge, Julia Wendt, Mathias Weymar, Alfons O. Hamm, Alexander Lischke

**Affiliations:** ^1^Department of Sport Sciences, University of Rostock, Rostock, Germany; ^2^Department of Orthopaedics, University Medicine Rostock, Rostock, Germany; ^3^Institute for Community Medicine, University Medicine Greifswald, Greifswald, Germany; ^4^Department of Psychology, University of Greifswald, Greifswald, Germany; ^5^Department of Psychology, University of Potsdam, Potsdam, Germany

**Keywords:** oral contraceptives, menstrual cycle, estrogen, progesterone, emotion recognition, social cognition

## Abstract

Despite the widespread use of oral contraceptives (OCs), remarkably little is known about the effects of OCs on emotion, cognition, and behavior. However, coincidental findings suggest that OCs impair the ability to recognize others’ emotional expressions, which may have serious consequences in interpersonal contexts. To further investigate the effects of OCs on emotion recognition, we tested whether women who were using OCs (*n* = 42) would be less accurate in the recognition of complex emotional expressions than women who were not using OCs (*n* = 53). In addition, we explored whether these differences in emotion recognition would depend on women’s menstrual cycle phase. We found that women with OC use were indeed less accurate in the recognition of complex expressions than women without OC use, in particular during the processing of expressions that were difficult to recognize. These differences in emotion recognition did not depend on women’s menstrual cycle phase. Our findings, thus, suggest that OCs impair women’s emotion recognition, which should be taken into account when informing women about the side-effects of OC use.

## Introduction

Although oral contraceptives (OCs) have been regarded as one of the best studied drugs in the history of medicine, remarkably little is known about the psychological and behavioral consequences of OC use (Montoya and Bos, 2017). Given that more than 100 million women worldwide use OCs for birth control (Christin-Maitre, 2013), studies investigating the effects of OCs on emotion, cognition, and behavior are highly warranted. Most relevant here, only a few studies investigated how OCs affect women’s ability to recognize other’s emotional expressions (Hamstra et al., 2014, 2015; Radke and Derntl, 2016). However, the ability to recognize others’ emotional expressions is essential for the initiation and maintenance of interpersonal relationships, in particular intimate ones (Schmidt and Cohn, 2001). As an inaccurate recognition of others’ emotional expressions may lead to interpersonal conflicts, it seems mandatory to further investigate how OC use affects women’s emotion recognition abilities. Previous studies revealed inconsistent findings regarding the effects of OCs on emotion recognition. Hamstra et al. (2014) coincidentally revealed a less accurate recognition of negative expressions in women who used OCs compared to women who did not use OCs. However, the impairments in emotion recognition partially depended on genetic variations of mineralocorticoid receptor haplotypes (Hamstra et al., 2015), complicating the interpretation of the respective findings. Radke and Derntl (2016), on the contrary, found no differences in emotion recognition between women with and without OC use. It should be noted, however, that methodological aspects of the studies, such as sample composition (e.g., inclusion of women at different menstrual cycle phases) or task characteristics (e.g., employment of tasks with suboptimal task difficulty), may have accounted for the inconsistency of findings. Consequently, it remains to be determined whether the coincidental findings of Hamstra et al. (2014, 2015) can be replicated and extended in studies that explicitly consider these methodological aspects throughout study design.

In the present study, we further investigated the effects of OC use on women’s emotion recognition abilities. As women with and without OC use may show minimal differences in emotion recognition (Radke and Derntl, 2016), we used a task that was sensitive enough to detect even subtle impairments in women’s emotion recognition (Reading the Mind in the Eyes Test, RMET; Baron-Cohen et al., 2001). The task required the recognition of complex emotional expressions, whereas the tasks of previous studies required the recognition of basic emotional expressions (Hamstra et al., 2014, 2015; Radke and Derntl, 2016). However, both types of tasks included expressions that had been categorized with respect to their valence. We were, thus, able to investigate whether differences in emotion recognition dependent on the valence of the expressions as suggested by Hamstra et al. (2014, 2015). As the expressions of our task had also been categorized with respect to their difficulty, we were able to investigate whether differences in emotion recognition dependent on the difficulty of the expressions as suggested by Radke and Derntl (2016). Accordingly, we performed two analyses to investigate differences in emotion recognition between women with and without OC use, one that was concerned with the valence of the expressions and another one that was concerned with the difficulty of the expressions. On basis of previous studies (Hamstra et al., 2014, 2015; Radke and Derntl, 2016), we expected that women with OC would show more impairments in emotion recognition than women without OC use, in particular during the processing of negative and difficult expressions. In addition to these hypothesis-driven analyses, we analyzed whether cyclic variations of women’s estrogen and progesterone levels contributed to possible differences in emotion recognition as suggested by Hamstra et al. (2014, 2015) as well as by Radke and Derntl (2016). It should be noted, however, that the respective analyses had an exploratory character because our study was designed to investigate differences in emotion recognition that dependent on women’s OC use rather than to investigate differences in emotion recognition that dependent on women’s menstrual cycle phase. However, combining exploratory with hypothesis-driven analyses allowed us to investigate the effects of OC use on women’s emotion recognition in greater detail than in previous studies (Hamstra et al., 2014, 2015; Radke and Derntl, 2016).

## Experimental Procedures

### Participants

Using G^∗^Power (Faul et al., 2007), we performed a power analysis to determine the number of participants that we needed to detect meaningful differences in emotion recognition between participants with and without OC use. Although Hamstra et al. (2014) found a large difference in emotion recognition between participants with and without OC use (*f* = 0.77–1.41), we based our power analysis on a more conservative estimate of the difference in emotion recognition (*f* = 0.30). To be able to detect a medium-sized difference in emotion recognition (α = 0.05, 1-β = 0.80, *f* = 0.30), we had to recruit a minimum of 62 participants for our hypothesis-driven analysis involving the comparison of women with and without OC use and a minimum of 75 participants for our exploratory analysis involving the comparison of women with OC use and women without OC use who were in the follicular or luteal phase of their menstrual cycle. In order to be considered for recruitment, women had to be aged between 18 and 35 years, to be native speakers and to be willing to share information regarding their menstrual cycle and their use of OCs (e.g., information regarding cycle length or OC type). Only women who had a regular cycle of 28 days and who, if at all, were not using any other hormonal contraceptives than oral ones were included in the study. Women who were in psychotherapeutic or psychopharmacological treatment were excluded from the study. Taking these inclusion and exclusion criteria into account, we recruited 95 women, 42 women with and 53 women without OC use, for our study. To determine the menstrual cycle phase of the 53 women who were not using OCs, we used a similar classification scheme as in previous studies (Derntl et al., 2008; Ertman et al., 2011). According to this classification scheme, 35 women without OC were in the follicular phase (0–14 days after menses onset) and 18 women without OC use were in the luteal phase (15–28 days after menses onset) of their menstrual cycle. More specifically, women without OC were either in the early follicular phase or in the mid-luteal phase of their cycle [follicular phase: *M* = 6.63, *SD* = 3.85, luteal phase: *M* = 20.11, *SD* = 4.08, *F*(1,51) = 139.86, *p* < 0.001, $\eta_{p}^{2}$ = 0.73]. Following an established procedure (DeBruine et al., 2005; Jones et al., 2005; Roney and Simmons, 2008), we used day-specific reference values of estrogen and progesterone levels that are typically displayed by women during the menstrual cycle (Stricker et al., 2006) to confirm that women without OC use were indeed in the follicular [estrogen (pg/ml): *M* = 77.82, *SD* = 63.96; progesterone (ng/ml): *M =* 0.28, *SD* = 0.18] or luteal [estrogen (pg/ml): *M* = 112.09, *SD* = 25.03; progesterone (ng/ml): *M* = 7.46, *SD* = 4.19] phase of their cycle [estrogen: *F*(1,51) = 4.76, *p* = 0.034, $\eta_{p}^{2}$ = 0.14; progesterone: *F*(1,51) = 104.27, *p* ≤ 0.001, $\eta_{p}^{2}$ = 0.67]. Of the 42 women who were using OCs, 21 were using OCs with androgenic properties and 21 were using OCs with anti-androgenic properties (see Table 1). As we were interested to investigate the global effects of OC use on emotion recognition, we recruited women who were in the inactive as well as active intake phase. All women provided written-informed consent before they participated in the study and were fully debriefed after they completed the study. The protocol of the study was approved by the ethics committee of the University of Rostock and the ethics committee of the German Society of Psychology (DGPs).

**Table 1:** Oral contraceptives.

Frequency	Compounds	Generation
2	EE (0.02 mg)/DRSP (3 mg)	4
5	EE (0.02 mg)/LNG (0.100 mg)	2
3	EE (0.02, 0.03 mg)/DSG (0.15 mg)	3
2	EE (0.02, 0.03 mg)/DRSP (3 mg)	4
4	EE (0.02, 0.03 mg)/LNG (0.100, 0.125 mg)	2
2	EE (0.02, 0.03 mg)/LNG (0.100, 0.150 mg)	2
3	EE (0.02, 0.03 mg)/LNG (0.100, 0.125, 0.150 mg)	2
5	EE (0.03 mg)/CMA (2 mg)	4
1	EE (0.03 mg)/CPA (2 mg)	4
11	EE (0.03 mg)/DNG (2 mg)	1
1	EE (0.03 mg)/LNG (0.125 mg)	3
2	EE (0.035mg)/NG (0.25 mg)	3
1	EE (0.035, 0.030, 0.035 mg)/DSG (0.05, 0.100 mg, 0.15 mg)	3

*EE, ethinylestradiol; CMA, chlormadinone acetate; CPA, cyproterone acetate; DNG, dienogest; DSG, desogestrel; DRSP, drospirenone; NG, norgestimate; LNG, levonorgestrel.*

### Procedure

Following a screening interview (Lischke et al., 2017), participants were invited to the laboratory where they completed a series of questionnaires regarding their menstrual cycle, contraceptive use, age, education, distress (Brief Symptom Inventory 18, BSI-18; Franke et al., 2017), and empathy (Interpersonal Reactivity Index, IRI; Davis, 1983). Thereafter, they completed the emotion recognition task (RMET; Baron-Cohen et al., 2001).

### Brief Symptom Inventory 18

The BSI-18 (Franke et al., 2017) was used to asses participants’ distress at the time of the study. The BSI-18, which measured anxious, depressive, and somatoform symptoms within the last 7 days, displayed good psychometric properties (BSI-18: α = 0.80).

### Interpersonal Reactivity Index

The IRI (Davis, 1983) was used to assess participants’ empathetic traits. The IRI, which measured empathetic traits related to empathetic concern, empathetic contagion, and empathetic perspective taking (Davis, 1994), demonstrated good psychometric properties (IRI: α = 0.76).

### Reading the Mind in the Eyes Test

The RMET (Baron-Cohen et al., 2001) was used to assess participants’ ability to recognize complex emotional expressions. These expressions had to be recognized on basis of subtle cues that were provided by the eye region of faces. The respective black and white pictures were shown in random order on a computer screen (1 practice picture, 36 test pictures). Each eye region was shown together with four labels, each describing a particular emotional expression (three distractors, one target). Participants had to indicate the label that best described the expression by pressing a corresponding button as fast as possible. On the basis of participants’ responses, the percentage of correctly identified expressions and the corresponding reaction times^1^ were measured. Similar as in previous studies (Guastella et al., 2010; Hysek et al., 2012; Feeser et al., 2015; Lischke et al., 2017; Lischke et al., 2018), established algorithms were used to determine these measures with respect to expressions that differed in valence (Harkness et al., 1999) and with respect to expressions that differed in difficulty (Domes et al., 2007).

### Statistical Analysis

SPSS 22 (SPSS Inc., Chicago, IL, United States) was used to run two sets of analyses, a hypothesis-driven one and an exploratory one. In the hypotheses-driven analyses, chi-square tests, one-way ANOVAs, and mixed-design ANOVAs were used to compare participant characteristics and emotion recognition performance between participants with and without OC use. In the exploratory analyses, chi-square tests, two-way ANOVAs, and mixed-design ANOVAs were used to compare participant characteristics and emotion recognition performance between participants with OC use, participants without OC use who were in the follicular phase, and participants without OC use who were in the luteal phase. The significance level for all analyses was set at *p* ≤ 0.05 (two-sided) and, whenever necessary, corrected for multiple comparisons using the Bonferroni method (Shaffer, 1995). However, the correction for multiple comparisons was only considered in the context of hypothesis-driven not exploratory analyses because exploratory analyses follow a liberal rather than conservative analysis strategy. In addition to the significance level, effect size measures (*d*, $\eta_{p}^{2}$) were reported to facilitate the interpretation of the respective findings (Cohen, 1992).

## Results

### Differences in Participant Characteristics

Chi-square tests and one-way ANOVAs revealed no differences in demographical [age: *F*(1,93) = 0.71, *p* = 0.401, $\eta_{p}^{2}$ = 0.01; education: χ^2^(1, *N* = 95) = 1.28, *p* = 0.259], psychopathological [distress: *F*(1,92) = 0.63, *p* = 0.439, $\eta_{p}^{2}$ = 0.01] or psychological [empathy: *F*(1,93) = 1.87, *p* = 0.175, $\eta_{p}^{2}$ = 0.02] characteristics between participants with and without OC use. Participants who used androgenic or anti-androgenic OCs also did not differ from one another with respect to their demographical [age: *F*(1,40) = 0.47, *p* = 0.495, $\eta_{p}^{2}$ = 0.01; education: χ^2^(1, *N* = 42) = 1.02, *p* = 0.311] psychopathological [distress: *F*(1,39) = 0.70, *p* = 0.407, $\eta_{p}^{2}$ = 0.02], or psychological [empathy: *F*(1,40) = 1.52, *p* = 0.226, $\eta_{p}^{2}$ = 0.04] characteristics. There were also no differences in demographical [age: *F*(2,92) = 0.62, *p* = 0.540, $\eta_{p}^{2}$ = 0.01; education: χ^2^(2, *N* = 95) = 1.28, *p* = 0.529], psychopathological [distress: *F*(2,91) = 2.29, *p* = 0.108, $\eta_{p}^{2}$ = 0.05], or psychological [empathy: *F*(2,92) = 1.97, *p* = 0.146, $\eta_{p}^{2}$ = 0.04] characteristics between participants with OC use and participants without OC use who were in the follicular or luteal phase of their menstrual cycle as indicated by chi-square tests and two-way ANOVAs. Taken together, these findings suggest that we investigated a sample of participants that was very homogenous in terms of demographical, psychopathological and psychological characteristics. A detailed account of these participant characteristics is given in Table 2.

**Table 2:** Participant characteristics.

	OC (*n* = 42)		OC-AP (*n* = 42)		OC-AAP (*n* = 42)		FOL+LUT (*n* = 53)		FOL (*n* = 35)		LUT (*n* = 18)	
	*M*	*SD*	*M*	*SD*	*M*	*SD*	*M*	*SD*	*M*	*SD*	*M*	*SD*
Age	22.55	2.45	22.81	2.66	22.29	2.26	23.08	3.42	22.86	3.49	23.50	3.33
Education												
Higher education	1		0		1		0		0		0	
Intermediate education	41		20		21		53		35		18	
Distress (BSI-18)^1^	0.47	0.32	0.43	0.30	0.52	0.33	0.53	0.39	0.60	0.42	0.40	0.32
Empathy (IRI)	50.77	7.98	50.05	8.19	46.90	8.35	48.48	8.33	51.91	7.64	48.56	8.40

*OC, women with OC use; OC-AP, women with androgenic OC use; OC-AAP, women with anti-androgenic OC use; FOL+LUT, women without OC use that were in the follicular and luteal phase; FOL, women without OC use who were in the follicular phase; LUT, women without OC use who were in the luteal phase; BSI-18, Brief Symptom Inventory 18 (Franke et al., 2017); IRI, Interpersonal Reactivity Index (Davis, 1983, 1994). ^*1*^Data were missing for one woman with OC use.*

### Valence-Dependent Differences in Emotion Recognition

**FIGURE 1:**
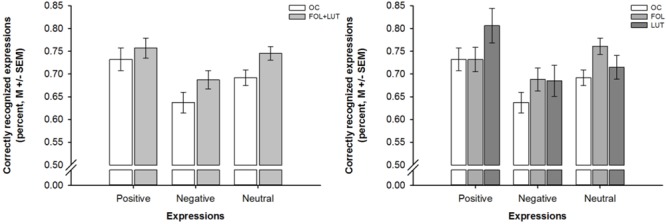
Barplots showing valence-dependent differences in emotion recognition as a function of OC use and menstrual cycle phase. *Left panel*. Valence-dependent differences in emotion recognition between women with OC use (OC, white bars) and women without OC use who were in the follicular and luteal (FOL + LUT, light gray bars) phase. *Right panel*. Valence-dependent differences in emotion recognition between women with OC use (OC, white bars) and women without OC use who were in the follicular (FOL, medium gray bars) or luteal (LUT, dark gray bars) phase. Bars represent *M* ± SEM.

A mixed-design ANOVA indicated that participants with OC use were less accurate in emotion recognition than participants without OC use [effect of group: *F*(1,93) = 6.51, *p* = 0.012, $\eta_{p}^{2}$ = 0.07; effect of valence: *F*(1.70,157.71) = 8.56, *p* = 0.001, $\eta_{p}^{2}$ = 0.08], irrespective of the expressions’ valence [interaction of group and valence: *F*(1.70, 157.71) = 0.29, *p* = 0.712, $\eta_{p}^{2}$ = 0.00]. Across all participants, recognition accuracy was lower for negative than positive or neutral expressions as indicated by *post hoc* tests [negative vs. positive: *p* = 0.003, negative vs. neutral: *p* = 0.002, positive vs. neutral: *p* = 0.608]. These differences in emotion recognition are shown in Figure 1.

Another mixed-design ANOVA revealed that the aforementioned differences in emotion recognition between participants with and without OC use did not depend on the menstrual cycle phase of participants without OC use [effect of group: *F*(1,92) = 3.28, *p* = 0.042, $\eta_{p}^{2}$ = 0.07; effect of valence: *F*(1.69,155.56) = 8.21, *p* = 0.001, $\eta_{p}^{2}$ = 0.08; interaction of group and valence: *F*(3.38,155.56) = 1.27, *p* = 0.288, $\eta_{p}^{2}$ = 0.03]. *Post hoc* tests indicated that participants with OC use were less accurate in emotion recognition than both, participants without OC use who were in the follicular phase [*p* = 0.035, *d* = 0.48] and participants without OC use who were in luteal phase [*p* = 0.038, *d* = 0.58]. There were, however, no differences in emotion recognition between participants without OC use who were in the follicular or luteal phase [*p* = 0.725, *d* = 0.10]. Again, these differences in emotion recognition occurred irrespective of the expressions’ valence because all participants were less accurate in the recognition of negative than positive or neutral expressions as indicated by *post hoc* tests [negative vs. positive: *p* = 0.001, negative vs. neutral: *p* = 0.003, positive vs. neutral: *p* = 0.111]. Figure 1 depicts the aforementioned difference in emotion recognition between participants with and without OC use who were in the follicular or luteal phase.

Of note, there were no differences in the recognition of positive, negative, or neutral expressions between participants who used OCs with androgenic and anti-androgenic properties (see the Supplementary Material). This implies that participants with OC use were generally less accurate in the recognition of these expressions than participants without OC use, irrespective of the type of OCs that was used by the participants.

### Difficulty-Dependent Differences in Emotion Recognition

**FIGURE 2:**
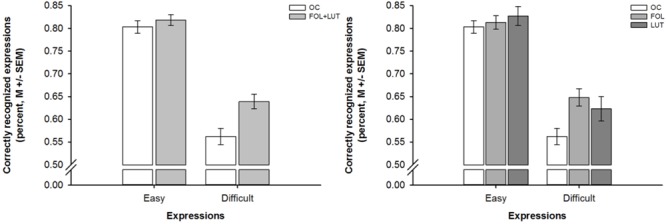
Barplots showing difficulty-dependent differences in emotion recognition as a function of OC use and menstrual cycle phase. *Left panel*. Difficulty-dependent differences in emotion recognition between women with OC use (OC, white bars) and women without OC use who were in the follicular and luteal (FOL+LUT, light gray bars) phase. *Right panel*. Difficulty-dependent differences in emotion recognition between women with OC use (OC, white bars) and women without OC use who were in the follicular (FOL, medium gray bars) or luteal (LUT, dark gray bars) phase. Bars represent *M* ± SEM.

A mixed-design ANOVA indicated differences in emotion recognition between participants with and without OC use that depended on the expressions’ difficulty [effect of group: *F*(1,93) = 7.52, *p* = 0.007, $\eta_{p}^{2}$ = 0.08; effect of difficulty: *F*(1,93) = 256.00, *p* = 0.001, $\eta_{p}^{2}$ = 0.73; interaction of group and difficulty: *F*(1,93) = 5.71, *p* = 0.010, $\eta_{p}^{2}$ = 0.06]. Follow-up ANOVAs revealed that these differences only emerged during the processing of difficult not easy expressions: Whereas participants with and without OC use did not differ in recognition accuracies for easy expressions [*F*(1,93) = 0.63, *p* = 0.428, $\eta_{p}^{2}$ = 0.01], participants with OC use were less accurate in the recognition of difficult expressions than participants with OC use [*F*(1,93) = 10.59, *p* = 0.002, $\eta_{p}^{2}$ = 0.10]. These differences in emotion recognition are depicted in Figure 2.

Another mixed-design ANOVA revealed that the aforementioned differences in emotion recognition between participants with and without OC use did not depend on the menstrual cycle phase of participants without OC use [effect of group: *F*(1,92) = 3.74, *p* = 0.027, $\eta_{p}^{2}$ = 0.08; effect of difficulty: *F*(2,92) = 214.51, *p* = 0.001, $\eta_{p}^{2}$ = 0.70; interaction of group and difficulty: *F*(2,92) = 3.41, *p* = 0.037, $\eta_{p}^{2}$ = 0.07]. Follow-up ANOVAs revealed again that participants with and without OC differed in recognition accuracies for difficult [*F*(2,92) = 5.53, *p* = 0.005, $\eta_{p}^{2}$ = 0.11] but not easy [*F*(2,92) = 0.47, *p* = 0.628, $\eta_{p}^{2}$ = 0.01] expressions. *Post hoc* tests indicated that participants with OC use were less accurate in the recognition of difficult expressions than both, participants without OC use who were in the follicular phase [*p* = 0.002, *d* = 0.74] and, albeit only on a trend level, participants without OC use who were in the luteal phase [*p* = 0.062, *d* = 0.53]. There were again no differences in emotion recognition between participants without OC use who were in the follicular or luteal phase [*p* = 0.471, *d* = 0.22]. These differences in emotion recognition are visualized in Figure 2.

Of note, participants who used OCs with androgenic and anti-androgenic properties did not differ in the recognition of easy and difficult expressions (see the Supplementary Material). This implies that participants with OC use were generally less accurate in the recognition of difficulty expressions than participants without OC use, irrespective of the type of OCs that was used by the participants.

## Discussion

In the present study, we investigated possible differences in complex emotion recognition between women with and without OC use. To this end, we administered a well-established emotion recognition task to a large and homogenous sample of healthy women who were well-characterized with respect to their OC use. We run two set of analyses, a hypothesis-driven one and an exploratory one. The hypothesis-driven analyses were used to test whether women with OC would be less accurate in emotion recognition than women without OC use and the exploratory analyses were used to explore whether these differences could be explained by cyclic variations of women’s progesterone and estrogen levels.

According to our hypothesis-driven analyses, women with OC use were less accurate in emotion recognition than women without OC use. Our findings are, thus, consistent with the findings of Hamstra et al. (2014, 2015) who found similar differences in emotion recognition between women with and without OC use. However, Hamstra et al. (2014, 2015) reported that these differences in emotion recognition were most pronounced during the processing of negative compared to positive expressions. We also observed that women with and without OC use tended to differ on the recognition of negative expressions (see Table 1), but our analyses indicated valence-unspecific rather than valence-specific differences in emotion recognition following OC use. Notably, Radke and Derntl (2016) failed to find any differences in emotion recognition between women with and without OC use, presumably because the task was not difficult enough to challenge women’s emotion recognition abilities. The task employed by Radke and Derntl (2016) involved the presentation of faces showing emotional expressions of maximal intensity, whereas the task employed by Hamstra et al. (2014, 2015) involved the presentation of faces showing emotional expressions of minimal, moderate, or maximal intensity. Consequently, women’s emotion recognition abilities were more challenged in the study by Hamstra et al. (2014, 2015) than in the study by Radke and Derntl (2016), thereby increasing the chance to detect even subtle differences in emotion recognition between women with and without OC use. The same may have been true for the task employed in the present study that involved the presentation of eye regions of faces expressing complex emotional expressions of varying intensities. In this respect, it is noteworthy that women with OC use showed the most pronounced impairments in emotion recognition during the processing of expressions that were difficult to recognize. It is, thus, quite likely that differences in task difficulty accounted for the inconsistent findings of previous studies (Hamstra et al., 2014, 2015; Radke and Derntl, 2016).

Our exploratory analysis revealed that women with OC use were less accurate in emotion recognition as both, women without OC use who were in the follicular phase and women without OC use who were in the luteal phase. Considering that OCs stabilize women’s menstrual cycle by suppressing the rise of gonadal hormones like estrogen and progesterone (Frye, 2006) may help to understand these differences in emotion recognition. Compared to women without OC use who are in follicular or luteal phase, women with OC use show much lower estrogen and progesterone levels (Fleischman et al., 2010). Low estrogen and progesterone levels may, thus, have been responsible for the impaired emotion recognition following OC use. However, estrogen and progesterone levels are also modulated by other hormones, implying that it may be too simplistic to assume that impairments in emotion recognition were caused by estrogen and progesterone alone. Oxytocin, for example, which is also affected by OC use, may interact with estrogen and progesterone during emotion processing (Lischke et al., 2012b; Scheele et al., 2016). It may, therefore, be more appropriate to assume that OC-induced impairments in emotion recognition are caused by various hormones, including but not limited to estrogen and progesterone.

However, estrogen and progesterone probably play a major role in mediating the effects of OC use on emotion recognition (Montoya and Bos, 2017). Previous studies revealed that estrogen and progesterone levels modulate activity and connectivity changes in prefrontal and temporal brain regions that are implicated in the processing of emotional expressions (Peper et al., 2011; Toffoletto et al., 2014). Of these brain regions, the prefrontal cortex and the amygdala may be of particular relevance because the recognition of complex emotional expressions crucially depends on activity and connectivity changes in these brain regions (Baron-Cohen et al., 1999; Adolphs et al., 2002; Adams et al., 2009; Dal Monte et al., 2014). OC-induced changes in estrogen and progesterone levels are associated with changes in amygdala activity and amygdala–prefrontal connectivity in the presence (Gingnell et al., 2013; Petersen and Cahill, 2015) and absence (Lisofsky et al., 2016; Engman et al., 2017) of emotion recognition tasks. It, thus, seems plausible that differences in amygdala activity and amygdala–prefrontal connectivity accounted for differences in emotion recognition between women with and without OC use.

Overall, our findings suggest that OCs impair the recognition of complex emotional expressions that are difficult to recognize, presumably via activity and connectivity changes in prefrontal and temporal brain regions that are caused by OC-induced changes in estrogen and progesterone levels. Although these suggestions are plausible, they should be treated with caution for several reasons. First of all, our study was designed to investigate differences in emotion recognition that dependent on women’s OC use rather than to investigate differences in emotion recognition that dependent on women’s menstrual cycle phase. As we were primarily concerned with the recruitment of women with and without OC use, we were unable to control for an unequal distribution of women across the different cycle phases. This may have been particularly problematic with respect to women who were in the luteal phase because these women were underrepresented in the present study. Nonetheless, we tried our best to characterize the women in the different cycle phases. As women’s self-reports may have been too inaccurate to determine their cycle phase, we tried to confirm their cycle phase on basis of their estrogen and progesterone levels. Similarly as in previous studies (DeBruine et al., 2005; Jones et al., 2005; Roney and Simmons, 2008), we used day-specific reference values for an estimation of women’s estrogen and progesterone levels during the respective cycle phases (Stricker et al., 2006). Following previous suggestions (Hamstra et al., 2014, 2015; Radke and Derntl, 2016), we used these hormone levels to investigate cycle-dependent differences in women’s emotion recognition. Clearly, it would have been favorable to use actual instead of estimated estrogen and progesterone levels in the respective analyses. Future studies should, therefore, assess these and other hormone levels via blood or salivary samples to further investigate how different hormones levels (e.g., estrogen levels, progesterone levels, or oxytocin levels) affect emotion processing in women with and without OC use. We, thus, labeled the respective analyses “exploratory” to highlight that the respective findings have to be replicated and extended in future studies. Second, our study was designed to investigate global rather than specific effects of OC use on women’s emotion recognition. Accordingly, we did not investigate whether the type (e.g., continued use, discontinued use), duration (e.g., short-term use, long-term use), or time (e.g., active use, inactive use) of OC use differentially affected the processing of emotional expressions in women. Future studies should, thus, gather more detailed information about women’s OC use than those that had been assessed in the present study. This may help to reveal more specific effects of OC use on women’s emotion recognition. These studies should also use emotion recognition tasks that allow a more specific assessment of the emotional expressions that are susceptible to OC effects. Whereas previous studies showed that women with OC use are impaired during the processing of negative expressions (Hamstra et al., 2014, 2015), the present study revealed that these impairments are most pronounced during the processing of expressions that are difficult to recognize. According to these findings, women with OC use may be specifically impaired during the processing of negative expressions that are difficult to recognize. Future studies that use more challenging emotion recognition tasks, like, for example, the morphed emotion recognition task (Lischke et al., 2012a), may help to identify emotion-specific impairments in women’s emotion recognition. These studies may also help to determine whether these impairments occur generally during the processing of expressions that are difficult to recognize, regardless whether these expressions are complex or basic ones. Third, our study was not designed to investigate the consequences of OC-induced impairments in women’s emotion recognition in interpersonal contexts. Future studies should, therefore, investigate whether these impairments alter women’s ability initiate and maintain intimate relationships. Fourth, our study was designed to investigate the effects of OC use on women’s emotion recognition in a quasi-experimental setting. Future studies that investigate these effects in experimental settings may be better suited to determine whether there is a causal or correlational relationship between OC use and impairments in emotion recognition.

To sum up, the findings of the present study suggest that OCs impair the recognition of complex emotional expressions that are difficult to recognize. However, these findings have to be extended and replicated in further studies before any recommendations about the current practice of OC use can be made. Considering that more and more women start using OCs shortly after onset of puberty (van Hooff et al., 1998; Krishnamoorthy et al., 2008) indicates that these types of studies are highly warranted to determine the positive and negative consequences of OC use on emotion, cognition, and behavior.

## Author Contributions

AL and RP designed the study, analyzed the data, and wrote the manuscript. AL, AM-M, JW, MJ, MW, and RP collected the data. AH, AM-M, JW, MJ, and MW contributed to writing, reviewing, and editing of the manuscript. All authors approved the final version of the manuscript.

## Conflict of Interest Statement

The authors declare that the research was conducted in the absence of any commercial or financial relationships that could be construed as a potential conflict of interest.

## Footnotes

1 In the following, we focus on participants’ recognition accuracy because the respective analyses did not reveal differences in reaction times that dependent on participants’ OC use or cycle phase (data not shown), implying that there was no speed accuracy trade-off that may have accounted for the reported differences in emotion recognition between participants with OC use and participants without OC use that were in the luteal or follicular phase of their menstrual cycle.
