# Supplementary material for: Oral Contraceptives Impair Complex Emotion Recognition in Healthy Women
Source: Front Neurosci. 2019 Feb 11;12:1041. doi: 10.3389/fnins.2018.01041 (PMC6378414; doi:10.3389/fnins.2018.01041)
Supplement: Supplementary file 1 [file Table_1.docx]

***Supplementary Material***

**Oral contraceptives impair complex emotion recognition in healthy women**

**Rike Pahnke^1^; Anett Mau-Moeller^1,2^, PhD; Martin Junge^3^, PhD; Julia Wendt^4^, PhD; Mathias Weymar^5^, PhD; Alfons O. Hamm^4^, PhD & Alexander Lischke^4^, PhD**

^1^Department of Sport Science, University of Rostock, Rostock, Germany
^2^Department of Orthopaedics, University Medicine Rostock, Rostock, Germany
^3^Institute for Community Medicine, University Medicine Greifswald, Greifswald, Germany
^4^Department of Psychology, University of Greifswald, Greifswald, Germany
^5^Department of Psychology, University of Potsdam, Potsdam, Germany

**Corresponding author:**

Alexander Lischke, Department of Psychology, University of Greifswald, Franz-Mehring-Str. 47, D-17487 Greifswald, Germany. Email: [alexander.lischke@uni-greifswald.de](mailto:alexander.lischke@uni-greifswald.de)

**Supplementary Material**

We performed several analyses to investigate whether there were any differences in valence- or difficulty-dependent emotion recognition between participants that used OCs with androgenic or anti-androgenic properties (see Table S1).

A mixed-design ANOVA revealed no differences in valence-dependent emotion recognition between participants that used androgenic or anti-androgenic OCs [effect of group: *F*(1,93) = 6.51, *p* = .012, *η^2^_p_* = 0.07; effect of valence: *F*(1.70,157.71) = 8.56, p *<* .001, *η^2^_p_* = 0.08; interaction of group and valence: *F*(1.70, 157.71) = 0.29, *p* = .712, *η^2^_p_* = 0.00, f = 0.05]. Across all participants, recognition accuracy was lowest for negative as compared to positive or neutral expressions as indicated by post hoc tests [negative vs. positive: *p* < .001; negative vs. neutral: *p* < .001; positive vs. neutral: *p* = .203].

There were also no differences in difficulty-dependent emotion recognition between participants that used androgenic or anti-androgenic OCs as indicated by another mixed design ANOVA [effect of group: *F*(1,93) = 7.52, p = .007, *η^2^_p_* = 0.08; effect of difficulty: *F*(1,93) = 256.00, *p* < .001, *η^2^_p_* = 0.73; interaction of group and valence: *F*(1,93) = 5.71, *p* = .010, *η^2^_p_* = 0.06]. Across all participants, recognition accuracy was lower for difficult than easy expressions.

As there were no differences in valence- or difficulty-dependent emotion recognition between participants that used androgenic or anti-androgenic OCs, we refrained from differentiating between these participants in our main analyses.

**Table S1**

*Emotion recognition*

|  | | OC-AP  (*n* = 21) | |  | OC-AAP  (*n* = 21) | |  |
| --- | --- | --- | --- | --- | --- | --- | --- |
|  | | *M* | *SD* |  | *M* | *SD* |  |
| Valence-dependent emotion recognition (RMET) | |  |  |  |  |  |  |
|  | Positive expressions | 0.73 | 0.17 |  | 0.74 | 0.13 |  |
|  | Negative expressions | 0.64 | 0.16 |  | 0.63 | 0.19 |  |
|  | Neutral expressions | 0.69 | 0.11 |  | 0.69 | 0.12 |  |
| Difficulty-dependent emotion recognition (RMET) | |  |  |  |  |  |  |
|  | Easy expressions | 0.78 | 0.10 |  | 0.82 | 0.08 |  |
|  | Difficult expressions | 0.58 | 0.10 |  | 0.54 | 0.14 |  |
| *Note.* OC-AP = women with androgenic oral contraceptive use, OC-AAP = women with anti-androgenic oral contraceptive use, RMET = Reading the Mind in the Eyes Test ([Baron-Cohen et al., 2001](#_ENREF_4)). | | | | | | | |
